# Impact of Coffee Bean Roasting on the Content of Pyridines Determined by Analysis of Volatile Organic Compounds

**DOI:** 10.3390/molecules27051559

**Published:** 2022-02-25

**Authors:** Marek Gancarz, Bohdan Dobrzański, Urszula Malaga-Toboła, Sylwester Tabor, Maciej Combrzyński, Daniel Ćwikła, Wacław Roman Strobel, Anna Oniszczuk, Hamed Karami, Yousef Darvishi, Alaksandra Żytek, Robert Rusinek

**Affiliations:** 1 Institute of Agrophysics Polish Academy of Sciences, Doświadczalna 4, 20-290 Lublin, Poland; m.gancarz@urk.edu.pl (M.G.); a.zytek@ipan.lublin.pl (A.Ż.); 2 Faculty of Production and Power Engineering, University of Agriculture in Krakow, Balicka 116B, 30-149 Krakow, Poland; umalagatobola@gmail.com (U.M.-T.); sylwester.tabor@urk.edu.pl (S.T.); 3 Pomology, Nursery and Enology Department, University of Life Sciences in Lublin, Głęboka 28, 20-400 Lublin, Poland; bdob@ipan.lublin.pl; 4 Department of Thermal Technology and Food Process Engineering, University of Life Sciences in Lublin, Głęboka 31, 20-612 Lublin, Poland; maciej.combrzynski@up.lublin.pl; 5 Rodzinna Palarnia Coffee and Sons Roastery, Boczna Lubomelskiej 4, 20-070 Lublin, Poland; daniel.cwikla@coffeeandsons.pl; 6 Institute of Technology and Life Sciences—National Research Institute, Falenty, Al. Hrabska 3, 05-090 Raszyn, Poland; w.strobel@itp.edu.pl; 7 Department of Inorganic Chemistry, Medical University of Lublin, Chodźki 4a, 20-093 Lublin, Poland; anoniszczuk@o2.pl; 8 Department of Biosystems Engineering, University of Mohaghegh Ardabili, Ardabil 56199-11367, Iran; hamedkarami@uma.ac.ir; 9 Department of Biosystems Engineering, University of Tehran, Tehran P.O. Box 113654117, Iran; sdarvishi@ut.ac.ir

**Keywords:** roasting coffee beans, convection–conduction, convection–conduction–radiation, VOCs, electronic nose, GC-MS, chemometrics

## Abstract

The aim of the study was to analyze the process of roasting coffee beans in a convection–conduction roaster (CC) without a heat exchanger and a convection–conduction–radiation roaster (CCR) with a heat exchanger for determination of the aroma profile. The aroma profile was analyzed using the SPME/GC-MS technique, and an Agrinose electronic nose was used to determine the aroma profile intensity. Arabica coffee beans from five regions of the world, namely, Peru, Costa Rica, Ethiopia, Guatemala, and Brazil, were the research material. The chemometric analyses revealed the dominance of azines, alcohols, aldehydes, hydrazides, and acids in the coffee aroma profile. Their share distinguished the aroma profiles depending on the country of origin of the coffee beans. The high content of pyridine from the azine group was characteristic for the coffee roasting process in the convection–conduction roaster without a heat exchanger, which was shown by the PCA analysis. The increased content of pyridine resulted from the appearance of coal tar, especially in the CC roaster. Pyridine has an unpleasant and bitter plant-like odor, and its excess is detrimental to the human organism. The dominant and elevated content of pyridine is a defect of the coffee roasting process in the CC roaster compared to the process carried out in the CCR machine. The results obtained with the Agrinose showed that the CC roasting method had a significant effect on the sensor responses. The effect of coal tar on the coffee beans resulted in an undesirable aroma profile characterized by increased amounts of aromatic volatile compounds and higher responses of Agrinose sensors.

## 1. Introduction

The mode of heat transfer in coffee bean processing has a considerable impact on the taste of brewed coffee [1,2]. In the coffee roasting process, heat can be transferred to coffee beans via three methods: convection, conduction, or radiation [3]. The most important variables for each type of roasting are the type of roaster, the temperature of the coffee beans, and the duration of the roasting process [4]. Convection heat transfer is based on hot air rising from warm surfaces towards coffee beans, whereas conduction heat is transferred via direct contact of the coffee beans with the warm surfaces of the rotating drum. Roasters based only on conduction heat do not ensure a homogeneous temperature distribution in the coffee beans due to their non-homogeneous contact with the drum surface in the roasting process. Heating via radiation is based on the emission of light and heat waves that penetrate the coffee bean; hence, very little energy is lost during the process, rendering this heating method the most efficient.

A problem in most roasters is the instability of the roasting process [3]. In fluidized-bed roasters, coffee beans “levitate” in a stream of hot gas, and the air velocity is difficult to control or cannot be controlled. Even in the most popular drum roasters, which lift and pass coffee beans through hot air, adjustment to one variable may cause large fluctuations in other parameters [5].

Convection–conduction–radiation (CCR) roasters differ from other roasting machines in the presence of burners with low nitrogen oxide emissions, which ensures very clean combustion. These roasters have over 50% higher efficiency than blue-flame burners, which substantially reduces energy costs and ensures much cleaner roasting. The CCR roaster method combines the most effective technologies, being based on several types of heating process. Heat exchangers provide the roasting process with clean hot air drawn via convection [6]. After the flame is turned off, the exchangers ensure a stable air temperature for 4–6 min. The carbon steel drum first radiates heat, which is dependent on the temperature of the metal and the conductivity coefficients of the coffee beans and the metal with which they come in contact. Next, the conductive heat acts via direct contact with the coffee beans. The CCR roaster operator can change the airflow rate by modifying the drum energy and temperature during the subsequent stages of the roasting process and depending on the size of the coffee bean batch. Therefore, CCR roasters can roast 250 g of coffee with the same precision as a full 90 kg batch. The independent control of convection and conduction heat and the precise control of energy transfer contribute to the maximization of the capacity of roasting profiles [7].

In contrast to CCR roasters, which do not use contaminated combustion gases as energy carriers [8], the method with combustion gases is still being used in convection–conduction (CC) devices, as in most drum roasters. However, in the coffee roasting process, such heat exchange has a considerable disadvantage. The ambient air is usually contaminated and saturates the coffee beans with nitrogen oxides, carbon oxides, carbon dioxide, and other gases that can make the taste of the coffee impure and less palatable [9,10].

Gas burners, which do not evenly distribute the heat around the drum leading to hot and cold spots, are still used in CC roasters. They do not ensure precise control of the airflow through the coffee bean-containing drum during the roasting process. This may result in various types of defect in the roasting process.

Several defects that may occur during the coffee roasting process yield baked, underdeveloped, overdeveloped, and scorched coffee beans [11,12,13]. The baked coffee defect occurs when coffee beans are heated too long at an insufficiently high air temperature without reaching the first crack. This defect, referred to as “stalling” the roast, is invisible but the roasted beans have a distinctive flat flavor with a slight sweetness, often described as a bread-like or papery flavor. Underdeveloped beans are usually grassy and devoid of caramelized sugars that are generated during the roasting process. This defect is usually observed when a light roast is carried out at a slightly lower than ideal temperature. Sometimes, but not always, the defect occurs when the roaster is set to light roast but its profile still needs slight adjustment. Overdevelopment of coffee is the opposite of underdevelopment. This defect occurs when the temperature is slightly too high for achievement of Vienna or French roasting. There is a subtle difference between the darker roast (Vienna, French) and overdeveloped coffee. The beans in both cases will look dark and greasy, sometimes even close to black, but the flavor of coffee brewed from overdeveloped beans will be burnt and bitter with smoky charcoal notes [14]. Scorched coffee is produced when the charge temperature, i.e., the initial temperature, is too high and the speed of the drum is too slow. This defect is reflected by dark burnt patches on the surface of the coffee beans, and the coffee tastes oily, smoky, and reminiscent of roasted poultry [14].

Given this information, it seems important to analyze the aroma of coffee roasted in CCR and CC roasters and correlate the presence of organic volatile substances (VOCs) as markers of coffee roasting defects.

Organic volatile compounds can be defined as a family of carbon-containing chemicals exhibiting high vapor pressure at ambient temperature. They have biological, chemical, or physical emission sources [15,16,17,18,19,20]. In coffee roasting, a combination of these processes is the emission source [21,22]. The decomposition of non-volatile compounds contained in raw coffee, pyrolysis, caramelization, and Maillard reactions contribute to the final composition of the coffee aroma [23,24]. The volatile fraction in roasted coffee is a highly complex mixture of different classes of compounds, where no single substance has the typical coffee flavor. Therefore, the aim of the study was to detect volatile organic compounds contained in five Arabica coffee varieties roasted in CCR and CC roasters and to identify any roasted coffee defects. The investigations were conducted with the use of the GC-MS SPME chromatographic technique [25,26] and an electronic nose with a measurement system based on a matrix consisting of eight metal oxide semiconductor (MOS) sensors [27].

## 2. Materials and Methods

### 2.1. Materials

The Arabica coffee beans originated from the five most important regions of coffee production: Costa Rica La Pastora, Ethiopia Sidamo, Brazil Santos, Peru El Palto Organic, and Guatemala Comal [21].

Costa Rica La Pastora—the ‘Caturra’ variety is grown in the San Marcos de Tarrazu region in Costa Rica. Ethiopia Sidamo—the ‘Heirloom’ variety is cultivated in the Sidamo region. Brazil Santos—the ‘Bourbon’ variety grows in lowlands. Peru El Palto—the ‘Caturra’ variety originates from the Amazonian Andes in Northern Peru. Guatemala Comal—the ‘Bourbon’ variety is grown in the area of San Pedro Necta commune.

The coffee beans were purchased from a professional wholesaler of green coffee in Poland (Rodzinna Palarnia Coffee and Sons Roastery).

### 2.2. Roasting Coffee

**Figure 1:**
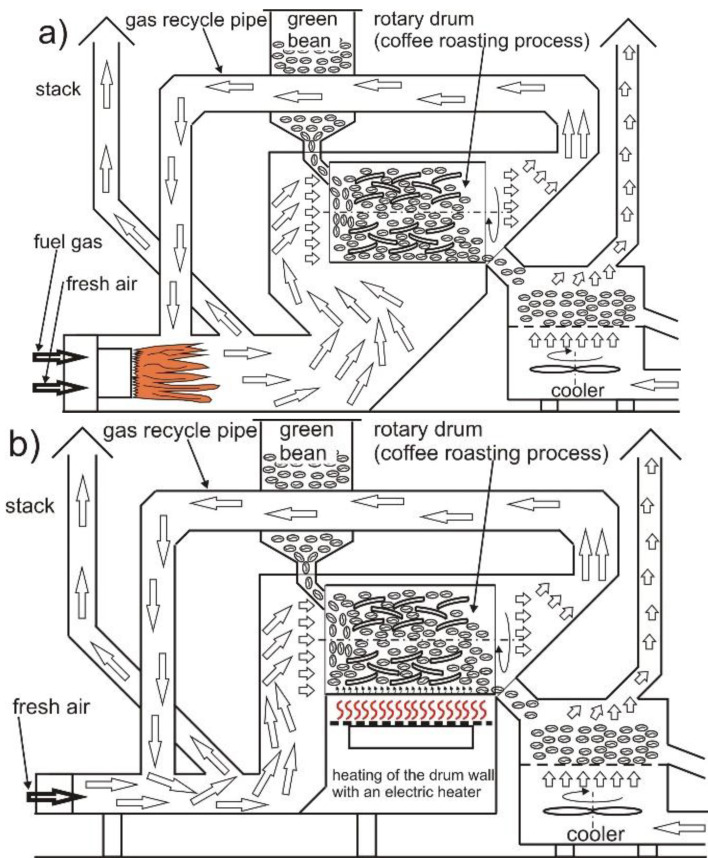
Scheme of the coffee roasting process in the convection–conduction roaster (CC)—(**a**); scheme of the coffee roasting process in the convection–conduction–radiation roaster (CCR)—(**b**).

The coffee beans were roasted in two types of roaster provided by well-known producers. In the present study, they are referred to as CCR and CC, which reflects the method of heat energy transfer (Figure 1). Based on preliminary tests, identical roasting parameters were selected for both roasters, with a standard burning time of 12 min 30 s, a drying phase of 6 min, a first roasting phase of 4 min, and a development phase of 2 min 30 s [13]. Each variant of coffee-roasting process was performed in three repetitions. The coffee been batches were identical in size, at 10 kg [21].

### 2.3. GC–MS Analysis

Volatile compounds were detected using a Trace GC Ultra gas chromatograph coupled with an ITQ 1100 mass spectrometer (ThermoFisher Scientific, USA) based on procedures developed previously [28,29].

The solid phase microextraction (SPME) technique was used to collect volatile compounds from the headspace above the coffee beans. To this end, an SPME fiber 50/30 µm Divinylbenzene/Carboxen/Polydimethylsiloxane (DVB/CAR/PDMS), Stableflex (2 cm) 24 Ga (Sigma Aldrich, Poland) was used.

The SPME fiber was placed in the Test Box model SR-3 (Figaro) measuring chamber for 30 min. The chamber contained volatile organic compounds emitted by the coffee beans. Next, the fiber was applied to the GC injector for 5 min to desorb VOCs. The injection port equipped with a 0.75 mm ID liner was maintained at 250 °C in the splitless mode. A Zebron ZB-5Msplus Capillary GC 30 m × 0.25 mm × 0.25 µm capillary column was used in the tests. The following parameters were used in the analysis: initial temperature of 60 °C maintained for 5 min, from 60 to 250 °C at 5 °C/min, from 250 to 270 °C at 10 °C/min, final temperature maintained for 5 min. The helium flow rate was kept constant at 2.2 mL/min. The temperature of the ion source transfer line was 280 °C. The electron ionization (EI+) mode with electron energy of 70 eV was applied. The mass spectrometer acquired data in the full scan mode (scan ranges: 35–390). Each variant of the experiment was performed in three repetitions. The procedure was also described in other publications by the authors of the present study [29,30].

### 2.4. Electronic Nose Analysis

An Agrinose electronic nose was used to determine the impact of volatile compounds on the receptors of chemically sensitive sensors, which are a substitute for the human olfactory apparatus [30,31,32,33]. It has a matrix of eight MOS sensors: TGS2600—general air contaminants, hydrogen, carbon monoxide; TGS2602—ammonia, hydrogen sulfide, high sensitivity to VOC and odorous gases; TGS2603—odors generated from spoiled foods; TGS2610—LP gas, butane; TGS2611—natural gas, methane; TGS2612—methane, propane, butane; TGS2620—solvent vapors, volatile vapors, alcohol; and AS-MLV-P2-CO—butane, methane, ethanol, hydrogen. The Agrinose has previously been used to evaluate bread spoilage, rancidity of edible oils, and seed spoilage, to control baking, and to distinguish between types of coffee. The measurement cycle and the sampling protocol consisted of a baseline purge for 10 s, a sample draw-in for 60 s, and a sample purge for 140 s. DasyLab software was used to convert analog signals to digital signals. The graph obtained was converted to the ∗.xls format and analyzed using statistical software. Each variant of the experiment was performed in three repetitions. The device and measurement procedures used are described in detail in other publications by the authors of the present study [30,34].

### 2.5. Statistical Analysis

Statistica software (version 12.0, StatSoft Inc., Tulsa, OK, USA) was used for statistical analyses. Principal component analysis (PCA), analysis of variance, and determination of correlations were performed at a significance level of α = 0.05.

The principal component analysis was employed to determine the relationships between the maximum responses of chemically sensitive sensors with the volatile compounds emitted from the coffee varieties under the two roasting methods [21]. The PCA data matrix for the statistical analysis of the results of the chromatographic tests had 21 columns (names of volatile compounds) and 30 rows (type of coffee and type of roast). In turn, the PCA data matrix for the statistical analysis of the results provided by the electronic nose had 8 columns (type of sensors) and 30 rows (max responses—ΔR/R_max_). The input matrix was scaled automatically. The optimal number of principal components obtained in the analysis was determined based on the Cattel criterion.

## 3. Results and Discussion

### 3.1. GC-MS Results

A number of changes correlated with the increasing temperatures that occurred during the roasting process in both types of coffee roaster [5]. The first drying stage proceeded in a temperature range of 20–130 °C. During this stage, the coffee beans became lighter. In the next stage (130–140 °C), the coffee beans began to swell and turned yellow due to the non-enzymatic browning process. The third stage (140–160 °C) was associated with a significant increase in the volume of the coffee beans and the number of micropores. Small cracks formed on the surface and the coffee beans became brittle. This was the first step in the generation of the aroma and volatile compounds. The subsequent step (160–190 °C) was characterized by progression of the Maillard reaction and other pyrolysis reactions into the internal dry structure of the coffee beans [23]. In the final stage (190–220 °C), the coffee beans emitted smoke and the volatilized carbon dioxide induced an increase in their porosity. The final stage yielded the typical coffee aroma produced by the main volatile compounds identified in the analysis [35,36,37].

The chromatographic analyses determined approximately 20 main volatile compounds (Table 1 and Figure S1). Different percentage contents were determined in the coffee beans originating from the five regions, but the major differences were revealed between the CCR and CC roasting methods.

In general, as shown by reports in the literature, over 800 volatile compounds can be determined in coffee beans, with approximately 20 compounds containing a pyridine ring, including methyl, ethyl, acetyl, and vinyl derivatives [37,38]. Pyridines are generated through trigonelline degradation reactions. They have an unpleasant plant-like and bitter odor that indicates their presence in products. For instance, 2-methylpyridine has a characteristic hazelnut odor, while 3-ethylpyridine has a buttery, plant-like, and caramel aroma. A stronger roasting degree is usually associated with higher pyridine content [13]. In the present study, the amount of pyridine was higher, which was probably related to the method of heat transfer to the coffee beans in the CC roaster rather than the roasting degree. The hot exhausts in the roaster without the exchanger had a direct impact on the coffee beans, inducing the generation of a higher content of pyridines through the activity of gas tar produced in the process of combustions of the fuel used to ensure high temperature in the roasting process. Another compound present in greater amounts in the Brazil Santos, Guatemala Comal, Costa Rica La Pastora, Ethiopia Sidamo, and Peru El Palto Organic coffee beans was 2-oxopropanal (aldehyde), which is a product of oxidation of primary alcohols. Aldehydes most often give coffee a note of fruit aroma and flavor. However, while the presence of, e.g., pyridine in the aroma profile of coffee beans is natural, increased amounts generated when roasting coffee bean in the CC roaster, relative to CCR, is regarded as a defect [14]. In excess it is harmful to the human organism and, in extreme cases, can lead to intoxication manifested by such symptoms as stupor, headache, nausea, and reduced appetite. Late symptoms include abdominal pain and pulmonary embolism. This chemical is also currently being investigated for its potential carcinogenicity. The available studies have not provided a convincing answer to the issue of probable carcinogenic effects of pyridine in the human organism; however, some tests carried out on animals confirm such an effect [12,39,40].

### 3.2. Electronic Nose Results

Table 2 shows the averaged values (based on three repetitions) with standard deviations (ΔR/R_max_) of the maximum responses of the chemically sensitive sensors to the aroma profile of the analyzed coffee beans roasted in the CC and CCR roasters.

The results obtained with the Agrinose showed an effect of the CC roasting method on the sensor responses. The presence of coal tar on the coffee beans resulted in an undesirable aroma profile characterized by increased amounts of aromatic volatile compounds [14]. The aroma in this case was more intense than in the CCR roasting process, hence the higher values of the responses of sensors AS-MLV-P2, TGS2600, TGS2602, TGS2610, TGS2612, and TGS2611. Sensors TGS2620 and TGS2603 responded in the opposite way. This was probably related to their sensitivity in detection of ethanol, which was more abundant in the aroma profile obtained in the CCR roaster (e.g., 2-furanmethanol and 2-buten-1-ol). The intensity of the VOC profile expressed by the ΔR/R_max_ parameter was more stable in the case of the CCR roasting method, and its values differed between the examined coffee varieties to a lesser extent [21]. This may indicate the repeatability of the roasting process in this type of roaster. The maximum response value in this case, i.e., ΔR/R_max_ = 2.92, was recorded for sensor TGS2602 (high sensitivity to low concentrations of odorous gases such as those generated from waste materials in office and home environments), and the lowest value, ΔR/R_max_ = 0.05, was noted for TGS2610 (very high sensitivity to LP gas). These values in the case of the CC roaster were ΔR/R_max_ = 3.81 for TGS2602 and ΔR/R_max_ = 0.06 for TGS2610, respectively.

### 3.3. Principal Component Analysis

**Figure 2:**
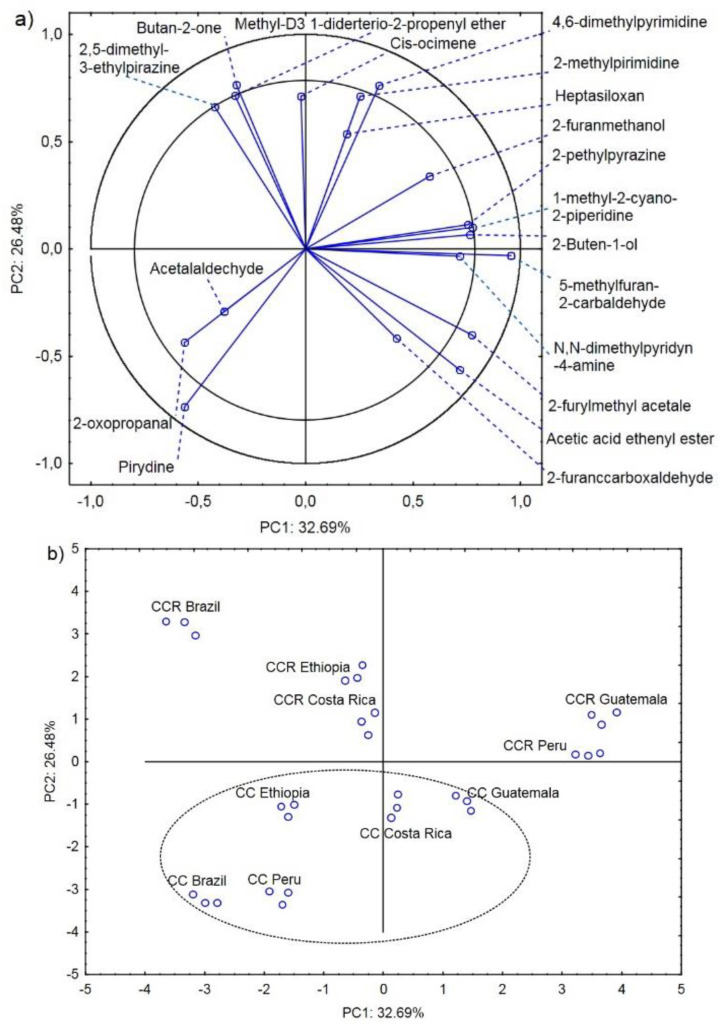
Projection of variables (main volatile compounds characterizing the CC and CCR coffee roasting process) on the PC1 and PC2 factor plane—(**a**); projection of cases (coffee beans from Brazil, Ethiopia, Guatemala, Costa Rica, and Peru roasted in the CC and CCR roasters) on the PC1 and PC2 factor plane—(**b**).

Figure 2a presents the projection of the variables on planes PC1 (32.69%) and PC2 (26.48%), which describe 59.17% of the relationships. A positive correlation was found between pyridine and 2-oxopropanal. There was also a strong negative correlation of these compounds with 4.6-dimethylpyrimidine and 2-methylpyrimidine. In turn, 2.5-dimethyl-3-ethylpirazine, butan-2-one, and methyl-D3 1-diderterio-2-propenyl ether exhibited a strong positive correlation. However, these compounds were strongly negatively correlated with another group of positively correlated compounds, i.e., 2-furylmethyl acetale and acetic acid ethenyl ester (Figure 2a).

Chemical compounds located in the area delimited by the two circles strongly influence the possibility of determination of the country of origin of the coffee beans together with the roasting process [21,23]. Butan-2-one, 2.5-dimethyl-3-ethylpirazine, methyl-D3 1-diderterio-2-propenyl ether, and methyl-D3 1-diderterio-2-propenyl ether characterized CCR Brazil, CCR Ethiopia, and CCR Costa Rica. 4.6-dimethylpyrimidine, 2-pethylpyrazine, 1-methyl-2-cyano-2-piperidine, 2-Buten-1-ol, and 2-methylpyrimidine characterized CCR Peru and CCR Guatemala. 5-methylfuran-2-carbaldehyde, 2-furylmethyl acetale, and acetic acid ethenyl ester characterized CC Guatemala and CC Costa Rica, and 2-oxopropanal and pyridine characterized CC Ethiopia, CC Peru, and CC Brazil. Acetalaldehyde, cis-ocimene, heptasiloxan, N.N-dimethylpyridyn-4-amine, and 2-furanccarboxaldehyde were a group of compounds that either did not differentiate or weakly discriminated the origin of the coffee beans and the roasting process type. Their percentage share in the coffee bean profiles was similar in all analyzed cases (Table 1).

Another important finding is that the compounds on the negative and positive sides of the PC2 principal component discriminated between the CC- and CCR-roasted coffee beans, respectively (Figure 2b). A particularly high amount of pyridine, acetic acid ethenyl ester, and 2-furylmethyl acetate is characteristic of the CC roasting process [38].

**Figure 3:**
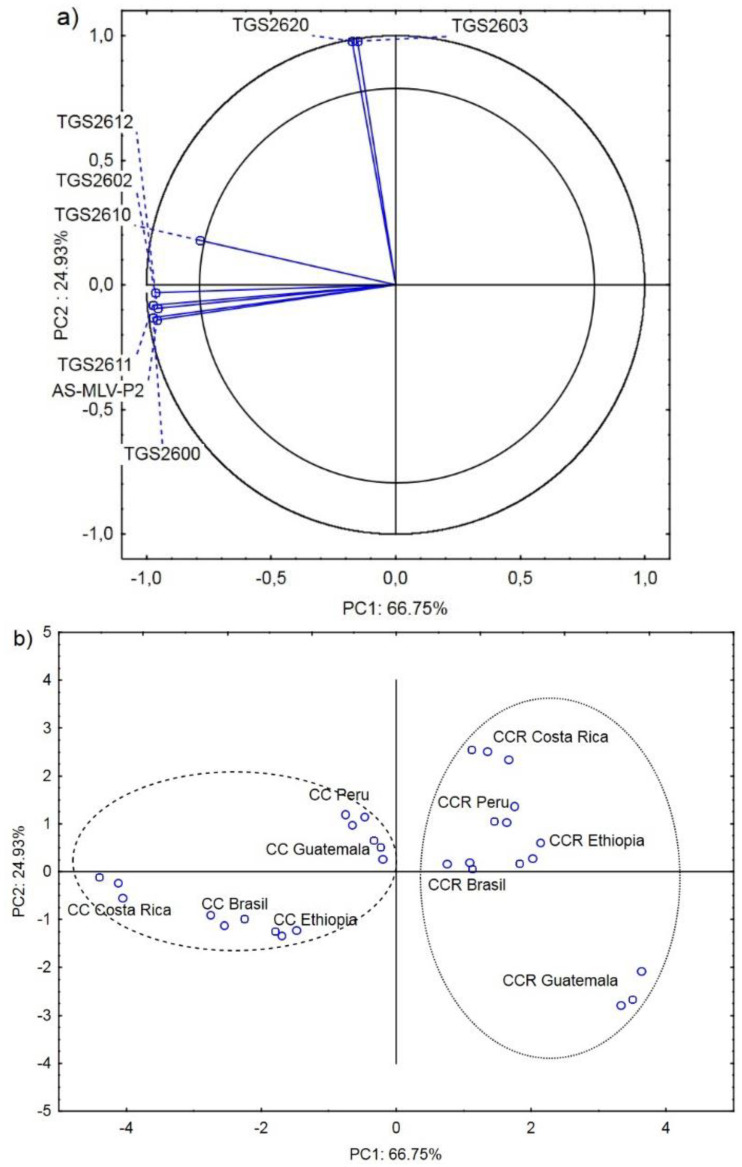
Projection of loadings (maximum responses of sensors characterizing the CC and CCR coffee roasting process) on the PC1 and PC2 factor plane—(**a**); projection of cases (coffee beans from Brazil, Ethiopia, Guatemala, Costa Rica, and Peru roasted in CC and CCR roasters) on the PC1 and PC2 factor plane—(**b**).

Figure 3a shows the projection of the variables (electronic nose response ΔR/Rmax) on planes PC1 (66.75%) and PC2 (24.93%). The analysis revealed a strong positive correlation between the responses of sensors AS-MLV-P2, TGS2600, TGS2602, TGS2610, TGS2611, and TGS2612. There was also a positive correlation between sensors TGS2603 and TGS2620. However, no correlation was found between these two groups (Figure 3a).

All Agrinose responses ΔR/Rmax for AS-MLV-P2, TGS2600, TGS2602, TGS2603, TGS2610, TGS2611, TGS2612, and TGS2620 located in the area delimited by two circles have a strong effect on the ability to distinguish the country of origin of the coffee beans and the roasting process, depending on the roaster type used [21]. The first principal component PC1 differentiates the volatile compound profile obtained in the roasting process in the CC roaster from that yielded by the CCR roasting process in as much as 66.75%.

Sensors AS-MLV-P2, TGS2600, TGS2611, TGS2610, TGS2602, and TGS12 most accurately characterized and described the CC roasting process’s generation of undesirable aromatic compounds associated with the influence of coal tar, which is a defect in this type of roasting process [13].

## 4. Conclusions

The process of roasting five coffee bean varieties from different regions of the world in the CC roaster was characterized by increased content of pyridines in the aroma of the coffee beans, in comparison with the CCR roaster equipped with the exchanger. This compound was generated in the process of combustion without the exchanger and was an effect of the presence of tar smoke on the coffee beans. The intensity of the aroma determined with the electronic nose and expressed by the maximum response of the chemically sensitive sensors was also higher in the case of the CC roasting process than in the case of the CCR roaster. This proves that the electronic nose is an excellent device for non-invasive and quick determination of coffee roasting conditions. In general, the CC coffee roasting process was associated with the adverse effect of the exhaust gases on the aroma. The aroma profile of the roasted coffee beans was not only a result of the roasting process and the roaster type, but was also dependent on the natural coffee bean profile determined by the growing conditions, i.e., the altitude of the plantation location, the terrain’s features, shading of the plantation, soil conditions, and the soil’s richness in mineral compounds. The aroma of coffee beans, which should be a result of species- and cultivation-related differences after the CC coffee roasting process, was dominated by the increased content of pyridine, which is a defect, compared to the CCR roasting process. These conclusions prove the presence of an undesirable defect in the coffee roasting process carried out without the use of a radiation heat exchanger. Coal tar produced through combustion of fuel providing high temperature in the roasting process exerts a negative effect on the aroma of roasted beans, which may adversely affect the flavor of brewed coffee.

## Figures and Tables

**Table 1:** Volatile compounds determined in the chromatographic analyses. %—percentage share of the compound in the tested sample immediately after CCR and CC roasting. Rt—retention time for Brazil Santos, Guatemala Comal, Costa Rica La Pastora, Ethiopia Sidamo, and Peru El Palto Organic coffee beans.

No.	Name	Chemical Formula	Rt	(%)									
				Brazil (CCR)	Brazil (CC)	Ethiopia (CCR)	Ethiopia (CC)	Guatemala (CCR)	Guatemala (CC)	Costa Rica (CCR)	Costa Rica (CC)	Peru (CCR)	Peru (CC)
1	2-Buten-1-ol	C_4_H_8_O	1.10	1.5	1.7	3.2	2.1	3.4	2.5	3.6	2.3	3.7	2.2
2	2-oxopropanal	C_3_H_4_O_2_	1.17	11.0	14.1	5.8	3.0	4.2	8.6	7.9	10.0	6.1	16.4
3	Methyl-D3 1-diderterio-2-propenyl ether	C_4_H_3_D_5_O	1.35	10.9	6.3	13.9	11.4	7.4	8.3	11.3	7.7	4.2	6.1
4	Acetalaldehyde	C_4_H_7_NO_3_	1.45	3.6	4.9	5.3	8.5	3.5	3.4	5.0	4.9	4.5	5.9
5	Pyridine	C_5_H_5_N	1.76	11.5	28.7	10.4	15.8	9.5	15.4	10.1	13.5	11.1	21.9
6	Butan-2-one	C_4_H_8_O	2.31	11.0	6.6	11.7	10.3	7.9	8.3	9.2	9.9	5.6	7.0
7	2-methylpyrimidine	C_5_H_6_N_2_	2.54	13.7	2.9	7.9	9.3	10.1	10.8	8.4	10.5	8.9	7.7
8	2-furancarboxaldehyde	C_5_H_4_O_2_	2.64	3.6	10.9	9.6	10.6	9.6	7.1	8.8	7.8	13.7	5.7
9	2-furanmethanol	C_5_H_6_O_2_	2.92	11.5	5.5	9.2	8.1	12.1	7.9	11.5	8.4	16.1	7.7
10	Acetic acid ethenyl ester	C_4_H_6_O_2_	3.16	2.5	3.2	3.2	3.5	4.0	3.9	3.9	3.8	4.7	4.2
11	4.6-	C_6_H_8_N_2_	4.25	8.9	5.5	6.4	6.2	8.8	7.6	6.1	6.9	6.3	4.4
	dimethylpyrimidine												
12	2-pethylpyrazine	C_6_H_8_N_2_	4.35	2.2	2.6	2.5	2.4	3.5	3.4	2.6	3.1	2.7	1.8
13	Cis-ocimene	C_10_H_16_	4.87	1.2	0.01	0.7	0.0	0.7	0.0	1.7	0.0	0.3	0.0
14	5-methylfuran-2-carbaldehyde	C_6_H_6_O_2_	5.78	1.6	1.7	3.3	3.2	4.7	3.7	3.6	3.8	4.8	2.6
15	2-furylmethyl acetal	C_7_H_8_O_3_	6.91	1.9	2.3	2.5	2.2	3.6	3.4	2.9	3.5	3.5	3.5
16	N.N-dimethylpyridyn-4-amine	C_7_H_10_N_2_	7.06	1.4	1.4	1.4	1.6	2.5	2.8	1.2	2.0	1.8	1.5
17	1-methyl-2-cyano-2-piperidine	C_7_H_10_N_2_	7.23	1.2	0.9	1.4	1.4	2.4	2.5	1.0	1.8	1.6	1.3
18	2.5-dimethyl-3-ethylpirazine	C_8_H_12_N_2_	9.73	11.5	0.6	0.3	0.3	1.0	0.3	0.2	0.1	0.2	0.0
19	Heptasiloxan	C_14_H_44_O_6_Si_7_	34.3	0.4	0.3	1.3	0.2	1.0	0.3	0.9	0.1	0.2	0.2

**Table 2:** Values of the maximum responses of chemically sensitive sensors to the intensity of aromas of Brazil Santos, Guatemala Comal, Costa Rica La Pastora, Ethiopia Sidamo, and Peru El Palto Organic coffee beans roasted in the CCR and CC roasters.

		ΔR/Rmax							
		AS-MLV-P2	TGS2600	TGS2602	TGS2610	TGS2612	TGS2611	TGS2620	TGS2603
**(CCR)**	Brazil	1.97 ± 0.07	1.37 ± 0.04	2.92 ± 0.05	0.07 ± 0.01	0.22 ± 0.02	0.19 ± 0.02	0.44 ± 0.03	0.41 ± 0.02
	Peru	1.85 ± 0.06	0.85 ± 0.05	2.80 ± 0.02	0.06 ± 0.01	0.22 ± 0.02	0.22 ± 0.02	0.47 ± 0.03	0.47 ± 0.02
	Guatemala	1.17 ± 0.03	0.57 ± 0.03	2.17 ± 0.03	0.05 ± 0.02	0.14 ± 0.01	0.15 ± 0.01	0.26 ± 0.02	0.26 ± 0.01
	Ethiopia	1.75 ± 0.03	0.90 ± 0.03	2.68 ± 0.03	0.07 ± 0.01	0.20 ± 0.02	0.19 ± 0.01	0.43 ± 0.02	0.42 ± 0.02
	Costa Rica	1.57 ± 0.04	1.00 ± 0.03	2.44 ± 0.04	0.13 ± 0.02	0.29 ± 0.02	0.24 ± 0.02	0.55 ± 0.02	0.53 ± 0.03
**(CC)**	Brazil	3.08 ± 0.03	2.11 ± 0.03	3.54 ± 0.04	0.15 ± 0.02	0.44 ± 0.02	0.41 ± 0.02	0.40 ± 0.01	0.38 ± 0.02
	Peru	2.38 ± 0.03	1.35 ± 0.02	3.21 ± 0.02	0.06 ± 0.01	0.25 ± 0.01	0.25 ± 0.01	0.48 ± 0.02	0.43 ± 0.03
	Guatemala	2.10 ± 0.01	1.56 ± 0.03	2.99 ± 0.03	0.10 ± 0.01	0.25 ± 0.01	0.24 ± 0.01	0.46 ± 0.01	0.42 ± 0.01
	Ethiopia	3.01 ± 0.02	2.08 ± 0.02	3.51 ± 0.01	0.12 ± 0.01	0.41 ± 0.01	0.37 ± 0.02	0.37 ± 0.02	0.37 ± 0.02
	Costa Rica	3.22 ± 0.02	2.59 ± 0.02	3.81 ± 0.02	0.31 ± 0.01	0.59 ± 0.02	0.50 ± 0.02	0.44 ± 0.02	0.43 ± 0.02

## Data Availability

Not applicable.

## Supplementary Materials

Figure S1: Sample chromatogram of Ethiopia coffee roasted with a CC oven, collected using the SPME technique.
