# Supplementary material for: Impact of Coffee Bean Roasting on the Content of Pyridines Determined by Analysis of Volatile Organic Compounds
Source: Molecules. 2022 Feb 25;27(5):1559. doi: 10.3390/molecules27051559 (PMC8911706; doi:10.3390/molecules27051559)
Supplement: Supplementary file 1 [file molecules-27-01559-s001.zip › molecules-1564469-supplementary.pdf]

Figure S1. Supplementary Materials

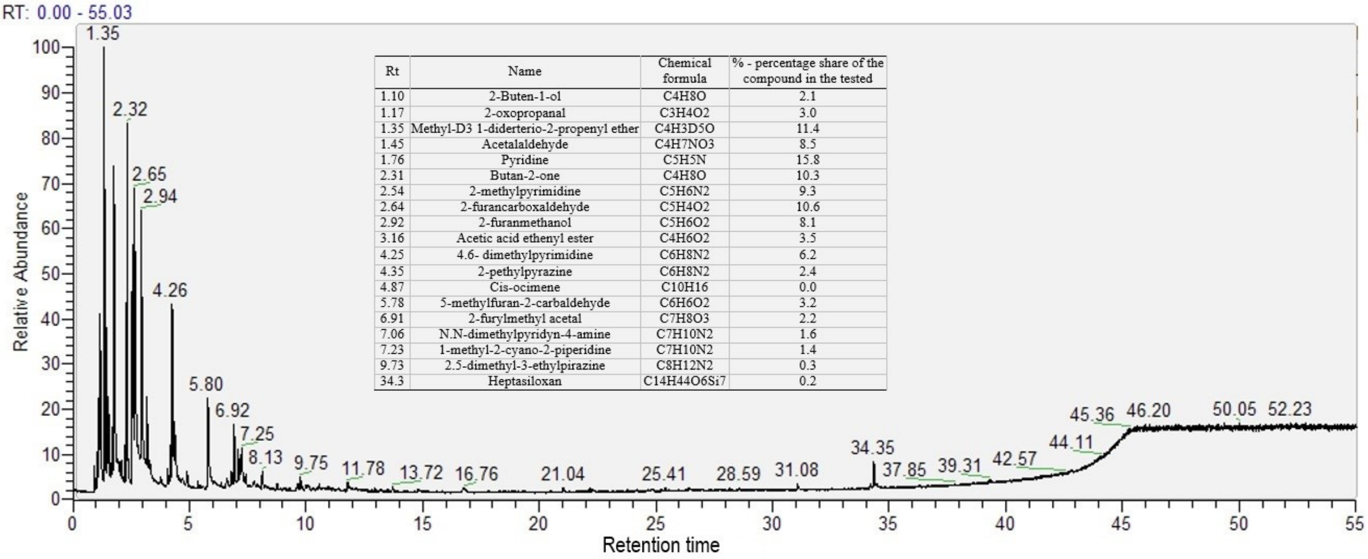

Rt	Name	Chemical formula	% - percentage share of the compound in the tested
1.10	2-Buten-1-ol	C4H8O	2.1
1.17	2-oxopropanal	C3H4O2	3.0
1.35	Methyl-D3 1-diderterio-2-propenyl ether	C4H3D5O	11.4
1.45	Acetalaldehyde	C4H7NO3	8.5
1.76	Pyridine	C5H5N	15.8
2.31	Butan-2-one	C4H8O	10.3
2.54	2-methylpyrimidine	C5H6N2	9.3
2.64	2-furancarboxaldehyde	C5H4O2	10.6
2.92	2-furanmethanol	C5H6O2	8.1
3.16	Acetic acid ethenyl ester	C4H6O2	3.5
4.25	4.6- dimethylpyrimidine	C6H8N2	6.2
4.35	2-pethylpyrazine	C6H8N2	2.4
4.87	Cis-ocimene	C10H16	0.0
5.78	5-methylfuran-2-carbaldehyde	C6H6O2	3.2
6.91	2-furylmethyl acetal	C7H8O3	2.2
7.06	N.N-dimethylpyridyn-4-amine	C7H10N2	1.6
7.23	1-methyl-2-cyano-2-piperidine	C7H10N2	1.4
9.73	2.5-dimethyl-3-ethylpirazine	C8H12N2	0.3
34.3	Heptasiloxan	C14H44O6Si7	0.2

Figure S1. Supplementary Materials. Sample chromatogram of Ethiopia coffee roasted with a CC oven, collected using the SPME technique.

Volatile chemicals were read from the chromatograms. Each peak seen in the graph at a given retention time corresponded to the intensity of the chemical that was identified from the Wiley 138 library.
